# The influence of the maternal microbiome on offspring neurodevelopment: a critical review of associations, controversies, and challenges

**DOI:** 10.3389/fnins.2025.1737795

**Published:** 2026-01-20

**Authors:** Hua Bai, Yan Xu, Shen Qu, Borui Li, Xinna Wang

**Affiliations:** 1 Bozhou Vocational and Technical College, Bozhou, China; 2 Affiliated Hospital of Changchun University of Chinese Medicine, Changchun, China

**Keywords:** association and causality, confounding factors, critical review, human milk, maternal gut microbiome, microbiota-gut-brain axis, neurodevelopment, prenatal programming

## Abstract

The role of the maternal microbiome in offspring neurodevelopment has become a prominent topic in neuroscience, yet its true causal status is under intense scrutiny. This critical review moves beyond conventional deconstructions of popular hypotheses in the field (e.g., “prenatal programming” “windows of opportunity”) to challenge a more fundamental premise. We systematically argue that the currently observed associations along the “microbiota-gut-brain axis” may reflect complex confounding, with macroscopic social factors such as socioeconomic status (SES) being the true underlying drivers. The core thesis of this paper is that the maternal microbiome is, to a great extent, a “biological imprint” of the mother’s living environment, diet, and stress levels—a highly sensitive “proxy” indicator acting as a biological mediator heavily shaped by the environment, rather than solely as an independent driver. By integrating evidence from social epidemiology, we contend that positioning the microbiome alongside factors like SES in a “flattened” network model is misleading. Instead, we propose a Hierarchical Causal Model where socioeconomic factors act as top-level “master regulators,” systematically shaping all downstream biological processes, including the microbiome. Through a critical analysis of interventions such as Fecal Microbiota Transplantation (FMT) and vaginal seeding, this review further exposes the translational predicaments that arise from neglecting this hierarchical structure. Ultimately, this review advocates for a paradigm shift: from searching for a single “microbial panacea” to understanding the microbiome’s true position within the socio-biological system, and proposes a conceptual framework for future research that is more aligned with real-world complexity and endowed with greater sociological imagination.

## 1 Introduction

The global burden of neurodevelopmental disorders is substantial and growing, presenting immense challenges to families and societies (Zhang et al., 2024). In the quest to understand their etiology, the scientific spotlight has shifted from traditional genetic determinism to the more complex interplay of genes and environment. For decades, research in social epidemiology has provided robust evidence that macro-environmental factors, such as socioeconomic status (SES), maternal stress, and childhood adversity, are among the strongest predictors of a child’s neurodevelopmental trajectory. As elucidated by the seminal work of Shonkoff et al. (2021) these social experiences become “biologically embedded” during development through profound physiological processes, shaping long-term health outcomes. With the rise of microbiome science in the 21st century, an intriguing candidate mediator for this “embedding” process has emerged. A powerful paradigm was proposed: the maternal gut microbiome, referred to as a “second genome,” may represent the critical biological link connecting the external environment to the developing offspring brain. This “microbiota-gut-brain axis” hypothesis posits that the gut microbiota engages in a bidirectional dialogue with the central nervous system via its metabolites, immune signals, and neural pathways, thereby influencing neurodevelopment (Cryan and Dinan, 2012). Extensive research in animal models has firmly established the biological plausibility of these pathways, demonstrating that microbial signals can indeed modulate neural outcomes under controlled conditions. However, amidst the enthusiastic exploration of these biological mechanisms, a fundamental question is often overlooked: to what extent do the observed “microbiome-neurodevelopment” associations reflect a direct causal effect of the microbiota, and to what extent are they merely a “biological echo” of more upstream, powerful socioeconomic factors?

While numerous epidemiological studies have revealed a robust association between breastfeeding and superior neurodevelopmental outcomes in offspring, efforts to attribute this link to specific microbial mechanisms face significant challenges and profound scientific controversy. The chasm between observed association and established causation is far wider than anticipated. This critical review, therefore, aims to judiciously evaluate the strength of existing evidence, clearly distinguish between causal evidence from animal models and correlational findings in human studies, and systematically explore alternative theories and knowledge gaps. We will focus on three core challenges. First, is the maternal microbiome a direct causal mediator driving offspring neurodevelopment, or merely a biomarker reflecting the mother’s overall health status? In human studies, disentangling the true effect of the microbiome from powerful confounders (e.g., SES, maternal diet, and mental health) is exceedingly difficult. Second, the physiological relevance in humans of the hypothesis that the maternal microbiota plays a role in programming fetal development prior to birth remains uncertain. Although trace amounts of microbial metabolites are detectable in cord blood, whether their concentrations are sufficient to exert a functional effect or are merely “background noise” from the maternal circulation is a point of intense debate. Third, during the postnatal period, the specificity of the effects of particular components in human milk, such as human milk oligosaccharides (HMOs), is also challenged. Are one or two “super-molecules” playing a decisive role, or is the network structure and overall functional output of the entire microbial ecosystem more important?

To address these challenges, this review will eschew a linear, deterministic narrative in favor of a critical evaluation framework.

To ensure a rigorous and comprehensive analysis, this critical narrative review synthesized literature searched across major databases, including PubMed, Web of Science, and Google Scholar, up to October 2025. We prioritized high-quality evidence, specifically longitudinal birth cohort studies, randomized controlled trials (RCTs), and mechanistic research that explicitly addressed confounding factors. Key search terms included “maternal microbiome,” “gut-brain axis,” “neurodevelopment,” “HMOs,” “socioeconomic status,” and “fecal microbiota transplantation.” Special attention was given to interdisciplinary literature at the intersection of social epidemiology and microbiome science to support the hierarchical model proposed herein.

The paper is structured as follows: Section 2 introduces the foundational knowledge of the microbiota-gut-brain axis and the bioactive components of human milk. Section 3, the core of the review, delves into the three aforementioned core challenges. Section 4 focuses on special populations, including preterm infants, cesarean-born infants, and children with neurodevelopmental disorders, to assess the true efficacy of interventional strategies. Section 5 and 6 systematically reflects on the fundamental difficulties in the field regarding model extrapolation, confounder control, and causal inference. Finally, in the integration and outlook of Section 7, we introduce a ‘Hierarchical Socio-Biological Causal Model.’ We explicitly contrast this new framework with previous ‘flat’ interaction models to guide the reader in understanding how socioeconomic factors act as upstream ‘master regulators’ that systematically shape the downstream microbiome.

While we emphasize the limited population-level impact of microbiome-targeted interventions under typical conditions, we acknowledge that such approaches may hold therapeutic promise in specific contexts. For instance, in high-risk subgroups—such as preterm infants, children with severe malnutrition, or those exposed to extreme environmental deprivation—microbiome modulation (e.g., via probiotics, prebiotics, or targeted nutritional support) could meaningfully alter developmental trajectories by mitigating acute biological disruptions. In these settings, the microbiome may function less as a passive proxy and more as a modifiable node within a compromised system. Thus, rather than dismissing microbiome-based strategies outright, our critique calls for precision: interventions should be grounded in clear mechanistic hypotheses, tested in well-defined populations, and evaluated against robust causal criteria.

## 2 Foundational concepts: basic theories and core mechanisms

### 2.1 The microbiota-gut-brain axis: five hypothesized communication pathways

The microbiota-gut-brain axis is conceptualized as a complex, bidirectional communication network. However, the specific pathways constituting this network and their relative importance in human physiology are largely hypotheses extrapolated from animal models and *in vitro* experiments. A clear understanding of the limitations of current evidence is crucial for a prudent assessment of research in this field. Currently, five main communication pathways are widely discussed in academia:

1. The Vagal Afferent Pathway Hypothesis: This hypothesis proposes that gut microbes can communicate with the brain via a relatively direct “neural circuit.” The posited mechanism involves sensory cells in the gut mucosa, such as enterochromaffin cells (ECs), which can sense microbial metabolites (e.g., SCFAs or tryptamine) and release neurotransmitters (e.g., 5-HT) (Kang et al., 2024). These chemical signals are then captured by the afferent fibers of the vagus nerve and transmitted to the nucleus of the solitary tract in the brainstem, subsequently influencing higher brain functions (Houser and Tansey, 2017). Key evidence supporting this hypothesis comes from a mouse study demonstrating that the anxiety-reducing effects of a specific probiotic strain (*Lactobacillus rhamnosus* JB-1) were completely abolished after vagotomy, suggesting that the vagus nerve is a necessary pathway for this strain’s action in this animal model (Bravo et al., 2011). However, the precise contribution of this pathway to the regulation of mood and cognition in humans remains unclear.
2. The Metabolite-Mediated Endocrine Hypothesis: This is currently the most extensively studied hypothesis. Its core idea is that small molecule metabolites produced by microbes can enter the bloodstream and act like hormones on distant organs, including the brain. Short-chain fatty acids (SCFAs) are the star molecules in this context, being highly concentrated in the human colonic lumen and detectable in peripheral blood (Mohajeri et al., 2018; Hou et al., 2024). In animal and cell models, SCFAs have been shown to possess multiple potential neuroregulatory functions. These include serving as an alternative energy source for the brain (den Besten et al., 2013), exerting epigenetic influence by inhibiting histone deacetylases (HDACs) (Jiang et al., 2021; Yu et al., 2020), and regulating the maturation and function of the brain’s resident immune cells, the microglia (Long-Smith et al., 2020). A landmark study in germ-free mice provided strong evidence for this: compared to normal mice, the microglia of germ-free mice exhibited immature morphology and function, and supplementation with SCFAs in drinking water alone could partially restore their normal state (Erny et al., 2015). Nevertheless, SCFAs must traverse the complex blood–brain barrier to get from peripheral blood to the brain parenchyma. Their exact concentration and functional contribution within the human brain remain actively investigated but far from resolved questions.
3. The Tryptophan Metabolism Axis Hypothesis: This hypothesis focuses on the metabolic partitioning of the essential amino acid tryptophan. The gut microbiome is a key regulator of tryptophan’s metabolic fate. It can promote the synthesis of over 90% of the body’s total peripheral serotonin (5-HT) in the gut, break down tryptophan into a series of neuroactive indole derivatives (e.g., indole-3-propionic acid, IPA), or influence host immune cells to direct it into the kynurenine (KYN) pathway (Wei et al., 2021). In animal models, these pathways have been shown to be closely related to neural function. For instance, a study in mice found that IPA produced by specific gut bacteria could cross the blood–brain barrier and exert anti-neuroinflammatory effects by activating the aryl hydrocarbon receptor (AHR) in astrocytes (Rothhammer et al., 2016). However, how these clear mechanisms observed in mice operate within the complex human system, and what the interactions and final balance point between different metabolic pathways are, remain significant unknowns.
4. The Barrier Integrity Modulation Hypothesis: This hypothesis posits that the health of the gut microbiome is intimately linked to the integrity of both the intestinal barrier and the blood–brain barrier. In theory, a healthy microbiota maintains the tight junctions of the intestinal barrier by producing substances like butyrate (Liu et al., 2005). Conversely, dysbiosis can lead to the leakage of inflammatory molecules like lipopolysaccharide (LPS) from Gram-negative bacteria into the bloodstream (i.e., “leaky gut”), which may then compromise the integrity of the blood–brain barrier, triggering or exacerbating neuroinflammation (Riedel et al., 2021; Kelly et al., 2015). Some animal studies support this model; for example, in obese mice induced by a high-fat diet, gut dysbiosis, blood–brain barrier damage, and cognitive decline were observed (Deng et al., 2025; Noble et al., 2017). It must be emphasized, however, that LPS concentrations in the blood of healthy humans are extremely low, and whether this is sufficient to have a significant impact on the blood–brain barrier in a non-infectious state, and the extent to which “leaky gut” is a cause rather than a consequence of neurodevelopmental disorders, remain widely debated.
5. The Neuroimmune Crosstalk Hypothesis: This hypothesis proposes that the gut microbiome systematically shapes the host’s immune status, which in turn affects the brain, by continuously “educating” the vast number of immune cells in the gut-associated lymphoid tissue (GALT) (Abo-Shaban et al., 2023; Kamada et al., 2013). In animal models, researchers have observed that immune cells “trained” in the gut (such as regulatory T cells, Tregs) can migrate to the central nervous system boundaries (e.g., the meninges) to exert local immunomodulatory effects (Fitzpatrick et al., 2020). The discovery of meningeal lymphatic vessels by the Kipnis team provided a revolutionary anatomical basis for direct dialogue between the peripheral and central immune systems, and suggested their function might be regulated by the gut microbiota (Louveau et al., 2015). However, the specific mechanisms of this pathway in human neurodevelopment and disease, especially in early life, are still largely speculative and lack direct human evidence.

### 2.2 Bioactive components in human milk: three key areas of research

Human milk is an exceedingly complex biological fluid that, in addition to basic nutrients, contains a vast array of potentially bioactive substances. Current research on these substances is mainly concentrated in the following three interconnected areas, but it is essential to recognize that their individual functions and interactions are still largely in the exploratory phase.

#### 2.2.1 Human milk oligosaccharides (HMOs): structural diversity and hypothesized functions

HMOs are the third most abundant solid component in human milk, after lactose and lipids. Their structures are extremely complex and diverse, with over 200 types identified to date (Konieczna et al., 2024). Their composition is significantly influenced by maternal genotype (e.g., the FUT2 gene), leading to distinct HMO profiles in the milk of different mothers (Durham et al., 2021; Spreckels et al., 2025). Based on *in vitro* experiments and animal models, researchers have proposed three major hypothesized functions for HMOs:

1. Selective Prebiotic Effect: HMOs are considered the “preferred food” for specific gut bacteria, particularly *Bifidobacterium longum* subsp. *infantis*, which possesses gene clusters for efficient HMO utilization (Underwood et al., 2015). However, it is noteworthy that the colonization rate of this strain in modern infants is declining, and other bacteria have also been found to partially utilize HMOs, suggesting complex functional redundancy and cross-feeding networks within the microbial community.
2. Anti-pathogen Adhesion: In *in vitro* experiments, free HMOs, due to their structural similarity to glycan receptors on the surface of intestinal epithelial cells, can act as “decoys” to bind with pathogens, thereby preventing them from attaching to the intestinal wall (Davis et al., 2016). The actual protective efficiency of this mechanism *in vivo* in infants has yet to be quantified.
3. Immunomodulatory Potential: Cell experiments indicate that some HMOs (especially sialylated HMOs) can interact with receptors on the surface of immune cells, thereby modulating immune responses (Tripp et al., 2024). Whether these *in vitro* observations translate into meaningful anti-inflammatory or immune tolerance-inducing effects in infants requires further investigation.

#### 2.2.2 The human milk microbiome: a field fraught with controversy

Whether a stable, functionally significant community of live bacteria exists in healthy human milk is a highly controversial topic. Although high-throughput sequencing can detect microbial DNA in human milk, with core genera typically including *Staphylococcus*, *Streptococcus*, and *Bifidobacterium*, the biomass is extremely low (approx. 10^3^–10^4^ CFU/mL) and highly susceptible to contamination from skin or the infant’s oral microbiota during collection (Spreckels et al., 2023). The origin of the milk microbiome is unclear, with two competing hypotheses: (a) the “entero-mammary axis” hypothesis, which suggests that maternal gut bacteria can be transported to the mammary gland via immune cells (Langel, 2025; Zhang et al., 2025); and (b) the “retrograde inoculation” hypothesis, which posits that microbes from the infant’s mouth enter the mammary gland during breastfeeding.

Given these uncertainties, the purported functions of the milk microbiome should also be considered as unverified hypotheses:

1. Direct Colonization Hypothesis: In theory, certain strains from human milk might successfully colonize the infant gut (Wu et al., 2025). However, strain-level tracking studies indicate that the true rate of mother-to-infant strain sharing may be much lower than expected, and many transmitted strains are only transient colonizers.
2. Immune Training Hypothesis: Microbes and their cellular components in milk (even dead bacteria) can act as antigens to gently stimulate the infant’s gut immune system, which is thought to aid in the establishment of oral tolerance (Perdijk et al., 2024).
3. *In Situ* Metabolism Hypothesis: Bacteria in milk may begin to ferment lactose or HMOs within the milk itself, producing metabolites like lactic acid (Kirmiz et al., 2018). However, whether the quantity of these *in situ*-produced metabolites is sufficient to have a physiological impact on the infant is unknown.

#### 2.2.3 Immunologically active factors: a protective network with more substantial evidence

Compared to the previous two areas, the functions of immunologically active factors in human milk are the most thoroughly studied and best-supported by evidence. Human milk is rich in various immunoglobulins (mainly secretory IgA, sIgA), antimicrobial proteins (e.g., lactoferrin, lysozyme), and immunomodulatory cytokines (e.g., TGF-β, IL-10) (Donovan, 2019). sIgA, produced by the maternal immune system, reflects the mother’s history of pathogen exposure and can bind to and neutralize pathogens in the infant’s gut, providing critical passive immune protection. It is also thought to play a role in shaping the composition of the early commensal microbiota (Hassiotou et al., 2013). Lactoferrin inhibits the growth of various bacteria by binding iron ions and has been suggested to selectively promote the proliferation of Bifidobacterium (Long-Smith et al., 2020). Anti-inflammatory cytokines like TGF-β and IL-10 are considered crucial for promoting Treg cell differentiation in the infant gut and establishing oral tolerance, which may have profound implications for preventing future allergic and autoimmune diseases (Kamada et al., 2013).

### 2.3 General developmental trajectory and high variability of the infant gut microbiome

The establishment of the infant gut microbiome is a dynamic and highly plastic process, not a fixed, linear program. Despite vast inter-individual differences, observations from large-scale longitudinal cohorts have delineated a general developmental trajectory from birth to 2 years of age. Understanding this trajectory and its key transition periods is crucial for exploring its potential synchrony with neurodevelopment.

#### 2.3.1 Phase I: approx. 0–3 months, stochastic colonization and environmental shaping

The neonatal gut is considered to have a very low microbial biomass at birth and is rapidly colonized by environmental microbes. The microbiota in this phase is typically dominated by facultative anaerobes that can tolerate micro-oxygenated environments, such as *Enterococcus*, *Escherichia*, *Staphylococcus*, and *Streptococcus* (Kamada et al., 2013). The source of these “pioneer” bacteria largely depends on the mode of delivery: vaginally born infants are primarily exposed to the maternal vaginal and gut microbiota (e.g., *Lactobacillus*), whereas cesarean-born infants are more exposed to maternal skin and hospital environment microbiota (e.g., *Staphylococcus*) (Dominguez-Bello et al., 2010). Consequently, the microbiota composition in this phase exhibits high stochasticity and environmental dependence.

#### 2.3.2 Phase II: approx. 3–12 months, breast milk-driven specialization

For breastfed infants, the gut microbiota undergoes a significant specialization process during this phase, characterized by the absolute dominance of the genus *Bifidobacterium*, especially subspecies that can efficiently utilize HMOs, such as *B. longum* subsp. *Infantis* (Underwood et al., 2015). The strong selective pressure exerted by HMOs in breast milk is considered the core driver of this shift. Functionally, gut metabolism in this phase is dominated by acetate production, which helps lower intestinal pH, inhibit pathogens, and may promote the maturation of the intestinal barrier. However, this “Bifidobacterium-dominant” pattern is not inevitable and its extent is influenced by various factors, including the composition of maternal HMOs and the infant’s colonization status with *B. infantis*.

#### 2.3.3 Phase III: approx. 12–24 months, transition to an adult-like pattern

With the introduction of solid foods and eventual weaning, the infant’s diet becomes increasingly complex, and the gut microbiome undergoes dramatic changes. The dominance of *Bifidobacterium* is gradually replaced by bacteria from the Firmicutes (e.g., *Faecalibacterium*) and Bacteroidetes phyla, which are capable of fermenting complex plant polysaccharides. The overall microbial diversity increases significantly (Myhill et al., 2020). Functionally, this means the production of short-chain fatty acids shifts from being acetate-dominant to a more diverse pattern with increased production of propionate and butyrate (Fawad et al., 2022; Duan et al., 2023). By around 2 years of age, the infant’s gut microbiome begins to resemble an adult-like pattern in composition and function. Some cohort studies have found an association between the “maturity” of the microbiome at this stage and the infant’s cognitive and motor development scores (Zimmermann et al., 2025), but this is merely a correlational observation, and the causal relationship and biological mechanisms remain unclear. We have summarized the system three critical developmental stages where the infant gut microbiome undergoes significant changes, potentially influencing neurodevelopment. Each period includes core features, health impacts, and current research status with associated challenges. The “window of opportunity” hypothesis suggests that certain microbial exposures during these periods may have a disproportionate impact on brain development. However, causal evidence is limited due to confounding factors (e.g., socioeconomic status), lack of replication, and insufficient mechanistic validation. Key research gaps include the need for longitudinal studies, causal modeling, and intervention trials to better understand these relationships. See Table 1 for details.

**Table 1:** Key transition periods and the “window of opportunity” hypothesis.

Key transition period	Core features and impacts	Research status and challenges
Delivery and the first week of life	Considered a critical period for initial microbial colonization, with the mode of delivery (e.g., vaginal vs. cesarean) exerting the strongest influence.	The impact of delivery mode on long-term health outcomes is well recognized. Further research is needed to clarify the exact mechanisms by which different delivery modes shape health trajectories.
Early infancy (approx. 1–6 months)	This is the key stage for breast milk-driven microbiota specialization. Compelling cohort studies have observed that the concentration of specific HMOs (e.g., 2′-FL) in breast milk during this period is significantly associated with cognitive scores at 2 years of age, while the association weakens later (e.g., at 6 months).	This has led to the hypothesis of a “window of opportunity” for the neurodevelopmental effects of HMOs. However, this observation still needs to be validated in more diverse populations, and its biological basis is far from being elucidated.
Weaning period (approx. 6–18 months)	This is the period of the most drastic remodeling of the microbiome, with a sharp increase in diversity and a fundamental shift in function.	A current research hotspot is whether disturbances during this period (e.g., antibiotic use, infections) can leave a long-term imprint on gut-brain axis function. More research is needed to determine the specific long-term impacts of these early-life perturbations and their potential reversibility.

HMOs, human milk oligosaccharides; 2′-FL:2′-fucosyllactose.

## 3 The eye of the storm: an in-depth analysis of core controversies

### 3.1 Challenge one: the enigma of the human milk microbiome’s contribution—direct seeding or indirect signaling?

Although the role of human milk in shaping the infant gut microbiome is undisputed, it remains uncertain whether live bacteria in milk directly colonize the infant gut or whether inactivated components are primarily responsible. This question is fraught with methodological challenges and continues to generate intense scientific debate.

#### 3.1.1 Evidence and limitations for the “direct seeding” hypothesis

The strongest evidence supporting the colonization of the infant gut by live milk bacteria comes from strain-level tracking studies using deep metagenomic sequencing. By comparing the whole-genome single nucleotide variation (SNV) patterns of shared strains in maternal milk and infant feces, these studies have confirmed the direct vertical transmission of specific strains (especially *Bifidobacterium* and some *Streptococcus*) from mother to infant (Wu et al., 2025). Some longitudinal studies have even observed a temporal sequence, where a specific strain is detected in the milk before appearing in the infant’s feces, providing evidence for the directionality of transmission (Stewart et al., 2018).

However, the practical significance of these findings is challenged by several key limitations. First, the efficiency and persistence of colonization may be overestimated. Earlier studies, or those using lower-resolution techniques (like PFGE and MLST), found that the true strain-level transmission rate might be only around 20–30%, and many successfully transmitted strains are merely “transient passengers,” showing only temporary colonization in the infant gut and disappearing rapidly after weaning (Davis et al., 2016; Sawhney et al., 2025). Second, a major and unavoidable confounder is retrograde contamination from the infant’s mouth. During breastfeeding, microbes from the infant’s oral cavity inevitably enter the mammary ducts and milk samples. A pivotal study comparing milk collected via direct breastfeeding with milk collected using a strict aseptic pump found that the latter had significantly lower microbial load and diversity, and its composition was much less similar to the infant’s oral microbiota (Al-Shehri et al., 2016). This strongly suggests that many signals previously attributed to the “milk microbiome” may actually originate from the infant itself.

#### 3.1.2 A more general alternative theory: indirect immunomodulation

Given the uncertainty of the evidence for direct seeding, a more general and methodologically robust alternative theory is gaining traction: the contribution of the milk microbiome is primarily achieved through indirect immunomodulatory signals, without the need for successful colonization by live bacteria. Evidence for this theory includes:

1. Immunological Activity of Non-viable Bacterial Components: Studies have found that even pasteurized human milk (which inactivates all live bacteria) can still partially promote the growth of beneficial gut bacteria (like *Bifidobacterium*) and affect the immune system when fed to infants (Fitzpatrick et al., 2020). This indicates that dead bacteria, cell wall components (like peptidoglycan, lipoteichoic acid), or their DNA can themselves act as a “microbial antigen library, “gently “training” the infant’s immune system by activating pattern recognition receptors (like TLRs) on the gut mucosa to induce tolerance to commensal bacteria.
2. Shaping Role of Milk Immune Factors: The sIgA antibodies in human milk can selectively bind to and clear potential pathogens in the infant gut while “sparing” beneficial commensals, thereby indirectly shaping the microbiota’s structure (Van de Perre, 2003). This process of “immune exclusion” does not depend on the transmission of live bacteria from the milk but is a form of “remote control” by the maternal immune system over the infant’s gut ecosystem.

#### 3.1.3 Integrated assessment: a shift from “whether” to “how important”

In summary, the focus of the debate on the human milk microbiome is shifting from “whether direct transmission exists” to “how large is its quantitative contribution and functional significance.” Although strain-level evidence indicates that limited, selective direct colonization does occur, its proportional contribution to the overall construction of the infant gut microbiome is likely much smaller than previously thought, and its direct causal effect on long-term neurodevelopment is currently entirely speculative. In contrast, the indirect immunomodulatory effects mediated by the abundant microbial components in milk, whether viable or not, appear to be a more reliable and universally applicable explanatory framework. However, it must be stressed that this framework itself is still a hypothesis, requiring more functional studies to validate how these immune signals ultimately translate into specific effects on neurodevelopment.

### 3.2 Challenge two: the window of opportunity for HMO neurodevelopmental effects lacks causal support and relies heavily on correlation

Some compelling human cohort studies have observed that the concentration of specific human milk oligosaccharides (HMOs), particularly 2′-fucosyllactose (2′-FL), in very early life (e.g., the first month) is significantly correlated with cognitive development scores in infants at 2 years of age, while this association weakens or disappears later (e.g., at 6 months) (Berger et al., 2020). This phenomenon has given rise to the hypothesis of a critical “window of opportunity” for the neurodevelopmental effects of HMOs. However, attempts to explain the biological basis of this phenomenon expose a typical dilemma in current research: the forced stitching together of findings from different evidence levels (human correlation, animal causality) to construct a logically plausible but actually very fragile causal chain.

A frequently cited, yet logically flawed, hypothetical model attempts to link the following three types of evidence, as shown in Table 2. By integrating these three lines of evidence, an appealing hypothesis emerges: early exposure to the human milk oligosaccharide 2′-fucosyllactose (2′-FL) promotes the growth of specific gut bacteria, which in turn increase the production of short-chain fatty acids (SCFAs); these metabolites may cross the blood–brain barrier and support myelination during a critical developmental window, ultimately contributing to enhanced long-term cognitive function. However, this is a classic logical fallacy, as it incorrectly equates correlation with causation and seamlessly connects findings from different species, study designs, and measurement metrics.

**Table 2:** Linking three lines of evidence: a hypothetical model under scrutiny.

Type of evidence	Description	Key study references
Human correlational evidence A	A correlation between early milk 2′-FL concentration and later cognitive scores in a human mother-infant cohort	Berger et al. (2020)
Human correlational evidence B	A correlation between total early milk intake and the degree of myelination in the brain’s white matter at age 7	Long-Smith et al. (2020)
Animal causal evidence C	Short-chain fatty acids (SCFAs, especially butyrate) produced by the gut microbiota are essential for the maturation of oligodendrocytes and myelination in a germ-free mouse model.	Erny et al. (2015)

2′-FL, 2′-fucosyllactose; SCFAs, Short-chain fatty acids.

#### 3.2.1 A critical deconstruction of the above hypothetical model

To understand the fragility of this hypothesis, we need to examine the multiple implicit, yet far from proven, assumptions behind it:

1. Assumption One: The “disappearance window” of HMO-metabolizing microbiota is the sole explanation.

1. This explanation posits that the “window of opportunity” exists because the “super-bacteria” capable of efficiently metabolizing HMOs (like *B. longum* subsp. *infantis*) decline in abundance after 6 months of age due to factors like the introduction of solid foods (Underwood et al., 2015). This is indeed a plausible speculation. But it ignores other possibilities: (a) Functional redundancy: Even if *B. infantis* disappears, other bacteria may take over some of the HMO metabolic functions (Davis et al., 2016). (b) Dilution effect of dietary substrates: After 6 months, dietary fibers from solid foods become a more significant source for SCFA production, which might render the SCFA contribution from HMOs less critical, thus “diluting” the unique effect of early HMOs.

1. Assumption Two: The “myelination sensitive window” of brain development is entirely dependent on microbial signals.

The brain does indeed have a burst of myelination in early life, and animal models suggest the importance of microbial signals (Erny et al., 2015). However, attributing the myelination of this window period entirely or primarily to SCFA signals from the gut is a gross oversimplification. Myelination is an extremely complex process co-regulated by genetics, maternal nutrition (e.g., DHA, choline), hormones, and many other factors. In human infants, what is the actual contribution percentage of gut-derived SCFAs to brain myelination? Is it a rate-limiting step? These key questions are complete unknowns.

1. Assumption Three: There is an irreversible epigenetic “memory window.”

Animal studies have shown that early microbial exposure can leave lasting “imprints” through epigenetic modifications (Brand et al., 2011; Martino and Prescott, 2010). Some probiotic intervention RCTs in preterm infants also suggest that the timing of intervention is crucial (Olsen et al., 2016). However, directly extrapolating these findings to the effect of HMO exposure in healthy term infants and asserting the existence of a “programming window” that cannot be reversed once closed lacks direct evidence.

#### 3.2.2 Integrated assessment: an intriguing observation, not a solved puzzle

In conclusion, the “time-window specificity” of HMO neuro-effects is an intriguing scientific observation based on high-quality human cohort studies. It provides a valuable entry point for exploring the complex relationship between early-life nutrition and brain development. However, all current explanations for its biological basis should be regarded as highly speculative hypotheses. The narrative that integrates gut microbiota succession, brain development tempo, and epigenetic plasticity is elegant but conceals the vast evidentiary gaps behind it.

More importantly, we must confront the potential internal logical contradictions within these explanations. For example, the alternative theory of the “dilution effect of dietary substrates,” while seemingly plausible, paradoxically weakens the independent importance of HMOs from another angle. If the introduction of solid foods can easily “dilute” or “override” the unique effects of HMOs, this itself implies that the signal from HMOs may be relatively weak and non-decisive. This contradiction precisely supports the hierarchical model proposed at the end of this review: the effects of biological factors like HMOs are likely strongly modulated by more macroscopic dietary patterns and environmental factors. Future research must commit to longitudinally and synchronously measuring all these variables in the same cohort using multi-omics approaches to have any chance of truly solving this puzzle, rather than continuing to build plausible but evidence-deficient “stories.”

### 3.3 Challenge three: prenatal microbial programming—a hypothesis unproven in humans

The “sterile womb” has been a traditional cornerstone theory in obstetrics and neonatology, positing that the fetus is entirely sterile in utero and microbial colonization begins during the birthing process (Dubé-Zinatelli et al., 2025). In recent years, however, this dogma has been challenged by the “prenatal microbial programming” hypothesis. This hypothesis suggests that even without live bacterial colonization, the maternal gut microbiota can influence fetal development across the placenta via its metabolites or immune signals. This section aims to critically examine this controversy and assess the strength of the evidence for this hypothesis in humans.

#### 3.3.1 The rise and fall of the “placental microbiome”: a cautionary tale of contamination

The first wave of challenges to the “sterile womb” began in 2014, when Aagaard et al. (2014) in a widely cited study, claimed to have found a unique, low-biomass microbiome in the human placenta. This finding quickly spurred a flurry of subsequent research, seemingly heralding the birth of a new field. However, the academic excitement was soon replaced by a series of rigorous methodological studies. By introducing stricter negative controls (including blank swabs from the collection process and DNA extraction from pure water) and advanced contamination filtering algorithms, these studies convincingly demonstrated that the so-called “placental microbial signals” in early studies were overwhelmingly derived from external contamination, including laboratory reagents, environmental DNA, and cross-contamination during delivery (Gao et al., 2025; Egan et al., 2021). Currently, the mainstream consensus in the academic community has returned to a more cautious position: in a healthy pregnancy, the placenta and amniotic fluid are functionally sterile, and any detected microbial DNA is more likely to be non-viable “molecular fossils” rather than evidence of live bacterial colonization.

#### 3.3.2 “Functional prenatal programming”: intriguing clues from artificial animal models

Although the hypothesis of prenatal live bacterial colonization has been largely refuted, a more subtle idea has taken its place: can the maternal microbiota “functionally program” the fetus through its small molecule products? The strongest evidence supporting this view comes from highly artificial germ-free animal models. In an elegant experiment, Vuong et al. (2020) transiently colonized germ-free mother mice with a single bacterial strain during pregnancy and then returned them to a germ-free state before delivery to ensure the offspring were sterile at birth. The results were striking: the brain development of these offspring during the embryonic stage (e.g., expression of axon growth-related genes) was already different from the completely germ-free control group, and the levels of specific tryptophan metabolites derived from the maternal microbiota in their blood were also altered (Kim et al., 2017). Other animal studies have also suggested that the maternal microbiota can indirectly affect the fetal immune and metabolic systems by influencing the mother’s own metabolic state (e.g., blood glucose, inflammation levels) (Kimura et al., 2020) or by inducing the transplacental transfer of IgG antibodies.

#### 3.3.3 From animal models to human reality: a vast, uncrossed chasm

Despite the appealing results of the animal experiments described above, extreme skepticism must be maintained when extrapolating them to humans. A core, unresolved issue is dose and functional relevance. Although studies can indeed detect trace amounts of microbial metabolites (like SCFAs) (Zhang et al., 2025) and T cells with a “memory phenotype” (Fitzpatrick et al., 2020) in human cord blood, their concentrations and numbers are extremely low. These trace signals confirm that a channel for maternal-fetal chemical communication exists. However, a critical question remains: are these low-abundance metabolites sufficient to drive neurodevelopmental programming in a complex human environment, or do they primarily reflect a sub-threshold resonance of the maternal circulatory state? This is a key question that no one can currently answer.

#### 3.3.4 Integrated assessment: an unresolved, open question

In summary, “prenatal microbial programming” is a highly inspiring scientific hypothesis that prompts us to rethink the biological dialogue between mother and fetus. However, we must be soberly aware that the status of this hypothesis in humans remains highly speculative and completely open. It is premature to dismiss the “sterile womb” dogma as “outdated.” A more accurate description is that the fetus is likely sterile in an anatomical and microbiological sense, but whether and to what extent it is exposed to functionally significant chemical or immune signals derived from the maternal microbiota remains a major, unresolved scientific question. Until more direct human functional evidence is obtained, any assertion that prenatal microbial programming determines the trajectory of human neurodevelopment lacks a solid scientific foundation.

## 4 Expanding the horizon: applications and challenges in special contexts

### 4.1 The vulnerability of preterm infants: a vicious cycle hypothesis of gut-brain injury

Preterm infants, especially very low birth weight infants, are at high risk for both necrotizing enterocolitis (NEC) and long-term neurodevelopmental impairment. The “gut-brain axis” hypothesis linking these two conditions is a focus of current neonatal research, but the causal relationships and the effectiveness of intervention strategies remain fraught with uncertainty.

#### 4.1.1 Typical dysbiosis pattern in the preterm infant gut microbiome

Compared to healthy term infants, the gut microbiome of preterm infants typically exhibits a dysbiotic pattern described as “three highs and one low”: (a) high proportion of Proteobacteria, especially potentially pathogenic *Escherichia coli* and *Klebsiella*; (b) high volatility, with an unstable microbiota structure; (c) high abundance of antibiotic resistance genes due to frequent antibiotic use; and (d) low abundance of beneficial bacteria, particularly *Bifidobacterium* and Bacteroides (Long-Smith et al., 2020). This dysbiotic pattern is considered the result of a combination of the preterm infant’s own physiological immaturity (e.g., insufficient gastric acid, slow intestinal motility) and the environmental pressures of the neonatal intensive care unit (NICU), especially antibiotic exposure.

#### 4.1.2 NEC-associated neurological injury: a warning from humanized animal models

NEC is one of the most fatal intestinal emergencies in preterm infants, with a mortality rate as high as 15–30% (Heath et al., 2019). Multiple studies have found that before the clinical symptoms of NEC appear, the infant’s gut microbiome undergoes drastic changes, typically characterized by a sharp expansion of Proteobacteria and a depletion of butyrate-producing bacteria (Underwood et al., 2015). To investigate the causal link between this dysbiosis and brain injury, an innovative study used a “humanized germ-free mouse” model. Researchers transplanted the fecal microbiota from an NEC infant before the onset of the disease into germ-free pregnant mice. Their offspring not only showed impaired intestinal barrier function but also significant neurodevelopmental abnormalities, including delayed myelination and anxiety-like behavioral deficits (Jiang et al., 2014; Chen et al., 2021).

However, extreme caution must be exercised in interpreting this striking result. The study was conducted in an animal model using a microbiota transplant from a single infant, and it remains highly uncertain whether the findings are generalizable or to what extent they can be extrapolated to human preterm infants. Nevertheless, the work offers a proof of concept for a hypothesized injury cascade: intestinal inflammation may trigger systemic inflammation, which in turn could disrupt the blood–brain barrier, leading to neuroinflammation and white matter injury.

#### 4.1.3 Potential and reality of intervention strategies: human milk and probiotics

In terms of intervention, the protective effect of breastfeeding has the strongest evidence. A large body of clinical observations and randomized controlled trials (RCTs) has shown that exclusive human milk feeding significantly reduces the risk of NEC (Ou et al., 2020; Quigley et al., 2019). Furthermore, among NEC survivors, breastfeeding is also associated with better long-term neurodevelopmental outcomes. Some neuroimaging studies further suggest a positive correlation between early-life human milk intake and brain structure and cognitive function in childhood. The mechanisms behind these associations are presumed to be related to HMOs, immune factors, and various bioactive substances in human milk, but the specific causal pathways are not yet clear.

In contrast, the evidence for using probiotics as a preventive measure is more complex. On one hand, a Cochrane systematic review including over 10,000 preterm infants found that supplementation with specific multi-strain probiotic formulations effectively reduced the incidence of NEC and all-cause mortality (Han et al., 2025), which is a major clinical advance. On the other hand, the evidence for whether these probiotic interventions translate into long-term neurodevelopmental benefits is less clear. For example, a study of preterm infants who received probiotic intervention found that survival free of major neurodevelopmental impairment was comparable between groups probiotics 281 (75.3%) vs. placebo 271 (74.9%) (Jacobs et al., 2017). This result highlights the current dilemma in research: even if an intervention successfully improves gut health (e.g., by reducing NEC risk), its impact on complex brain development may be weak, difficult to detect, or masked by other more powerful factors (such as socioeconomic environment, family support).

### 4.2 The “microbial deficit” of cesarean section: a weak association riddled with confounders

With the global cesarean section rate continuing to rise (Singh et al., 2024), its long-term health effects on offspring are of great concern. Cesarean delivery bypasses the natural inoculation of vaginal microbes, leading to a “developmentally delayed” pattern in the neonatal gut microbiome, characterized by delayed colonization of *Bifidobacterium* and a lack of typical vaginal microbiota (e.g., *Lactobacillus*) (Dominguez-Bello et al., 2010). However, whether and to what extent this early “microbial deficit” translates into long-term neurodevelopmental risk is a controversial issue plagued by numerous confounders.

#### 4.2.1 Contradictions and uncertainties in epidemiological associations

Some large-scale birth cohort studies have indeed observed a weak association between cesarean section and the risk of neurodevelopmental disorders. For example, the Canadian CHILD cohort study suggested that cesarean delivery might be associated with a slight increase in the risk of ADHD at age 5 (Wang et al., 2024). However, the strength of this association was significantly attenuated after adjusting for a range of socioeconomic and family environmental factors.

This inconsistency in research findings strongly points to the key role played by powerful confounding factors. The factors that determine whether a cesarean section is performed (i.e., the indications for C-section, such as fetal distress, maternal pregnancy complications, advanced maternal age, etc.) may themselves be the root causes of differences in offspring neurodevelopment. Furthermore, maternal education level, family income, prenatal anxiety status, and postnatal feeding practices are all closely related to both the mode of delivery and child development outcomes. In a mediation analysis attempting to clarify these relationships, researchers found that only about 25% of the weak negative impact of cesarean section on infant socio-emotional development could be explained by differences in the gut microbiome. This finding precisely illustrates that even if the microbiome plays a role, it is far from the whole story, and may not even be the main part.

#### 4.2.2 The attempt and disillusionment of “vaginal seeding”

To compensate for the “microbial deficit” caused by cesarean section, an intervention called “Vaginal Seeding” once generated widespread interest. This method attempts to mimic the microbial exposure of natural birth by swabbing the mother’s vaginal fluids onto the mouth, nose, and skin of the C-section newborn. However, the initial optimism was quickly dampened by subsequent, more rigorous randomized controlled trials (RCTs). A key RCT found that the effect of vaginal seeding was very limited: it only transiently and partially altered the infant’s skin microbiome, with almost no long-term impact on the more important gut microbiome (Song et al., 2021).

More concerning are the potential safety risks. Since pregnant women may carry asymptomatic pathogens in their vaginal tract (such as Group B Streptococcus, herpes simplex virus), this procedure carries the risk of directly transmitting pathogens to newborns with immature immune systems. Given its unclear efficacy and real risks, major professional authorities worldwide, including the American Academy of Pediatrics (AAP) and the European Society for Paediatric Gastroenterology, Hepatology and Nutrition (ESPGHAN), have issued clear guidelines against recommending vaginal seeding as a routine clinical practice (Limaye and Ratner, 2020). Instead, these guidelines emphasize promoting strategies that are proven to be safe and effective, such as encouraging early mother-infant skin-to-skin contact, exclusive breastfeeding, and avoiding unnecessary antibiotic use, to support the establishment of a healthy gut microbiome in all infants.

### 4.3 The gut-brain axis hypothesis in ASD and ADHD: the difficult leap from biomarker to intervention target

The gut-brain axis hypothesis in Autism Spectrum Disorder (ASD) and Attention-Deficit/Hyperactivity Disorder (ADHD) is one of the most fascinating and controversial topics in neuroscience today. The hypothesis posits that dysbiosis of the gut microbiome plays a significant role in the pathophysiology of these conditions. However, the leap from using the microbiome as a biomarker of disease to viewing it as an effective intervention target is far more difficult than anticipated.

#### 4.3.1 Microbiome association features in ASD and ADHD

Numerous case–control studies and several meta-analyses have indeed revealed some consistent features in the gut microbiome of children with ASD that differ from healthy controls. These mainly include: reduced overall diversity, decreased abundance of certain beneficial bacteria (like *Bifidobacterium*), and increased abundance of certain *Clostridium* species. In ADHD, although research is less extensive and results are more heterogeneous, some studies have also suggested that a reduction in butyrate-producing bacteria (like *Faecalibacterium*) may be associated with the disorder (Stewart et al., 2018). These findings, supplemented by animal experiments showing that transplanting fecal microbiota from children with ASD to germ-free mice can partially replicate their behavioral phenotype, form the basis of the hypothesis. However, all this evidence is essentially correlational. It cannot answer a key question: is microbial dysbiosis a cause of ASD/ADHD, or merely a consequence of the disease state (e.g., picky eating, stress)?

#### 4.3.2 Clinical trials of fecal microbiota transplantation (FMT): the stark contrast between Hope and reality

The most direct attempt to target the microbiome for intervention is Fecal Microbiota Transplantation (FMT). In 2017, a pioneering open-label trial by Kang et al. ignited hope across the field. The study reported that after an 8-week microbiota transfer therapy in 18 children with ASD, both their gastrointestinal symptoms and core ASD behavioral symptoms showed significant and lasting improvement (Hung and Margolis, 2024). This stunning result quickly garnered immense attention from the media and patients’ families.

However, scientific progress relies on reproducibility and more rigorous validation. Subsequently, a smaller but more rigorously designed double-blind, placebo-controlled randomized controlled trial (RCT) delivered a more sobering result. This study found that, compared to the placebo group, children with ASD who received FMT was safe, but did not induce symptom relief at 12 weeks compared with placebo (Aroniadis et al., 2019).

This stark contrast, from the great success of an open-label trial to the muted outcome of a double-blind RCT, is a reality that the field must confront. It highlights the profound challenges facing FMT in the treatment of ASD, with possible underlying reasons including: (1) the vast heterogeneity of donor microbiota and transplantation protocols; (2) the high heterogeneity of ASD itself in etiology and clinical presentation; and (3) a more fundamental possibility—that for most children with ASD, gut microbial dysbiosis may not be a driving factor of their core pathology. Therefore, simply “correcting” the microbiota itself is not enough to reverse established and stable neural circuit abnormalities.

#### 4.3.3 Integrated assessment: a therapeutic direction still at the proof of concept stage

Currently, the clinical evidence for both FMT and commercially available “psychobiotics” in improving the core symptoms of ASD or ADHD is very weak or contradictory (Berger et al., 2020; Kwak et al., 2023). Targeting the microbiome for these complex neurodevelopmental disorders remains an exploratory direction at an early proof-of-concept stage, far from being ready for routine clinical application.

Based on the highest quality evidence available (i.e., double-blind RCTs), it cannot be concluded that FMT is effective for improving the core symptoms of ASD. The existence of a so-called “microbiome-responsive” subgroup is itself a new hypothesis to be tested, not a reasonable explanation for the current negative results. This kind of argument, which resorts to the “future possibility of discovering a responsive subgroup” in the face of negative results, risks “moving the goalposts” and is a logical pitfall to be wary of in scientific exploration. Future research must return to basics. Instead of rushing to develop “engineered probiotics” or promote FMT, the more urgent tasks are: (1) to clarify the temporal sequence and causal relationship between microbial dysbiosis and disease onset through large-scale, longitudinal, prospective cohort studies, and to test the “proxy hypothesis”; (2) to use multi-omics technologies to find reliable biomarkers that can distinguish different ASD/ADHD subgroups, while soberly recognizing that these biomarkers may merely be correlates of the disease rather than causal targets. Only after truly understanding the exact position of the microbiome in the hierarchical causal network of the disease can so-called “precision microbiome medicine” potentially transform from a beautiful vision into a clinically meaningful therapeutic strategy.

## 5 Limitations and critical perspectives: examining the field’s fundamental challenges

In the enthusiasm for exploring the link between the maternal–infant microbiome and neurodevelopment, we must maintain scientific prudence and confront three fundamental challenges facing the current research landscape. These challenges systematically limit our ability to draw definitive causal conclusions from existing data.

### 5.1 Challenge one: a cognitive upgrade from “confounding factors” to “common causes”

In all human observational studies, a core and almost perfectly unsolvable problem is how to properly understand the role of factors like socioeconomic status (SES), maternal health, and lifestyle. Traditionally, these factors are treated as Confounding Factors that need to be statistically “controlled” for. However, this view may severely underestimate their true status. Within the framework of epidemiological causal inference, when a variable is a common cause of both the exposure (e.g., microbiome) and the outcome (e.g., neurodevelopment), treating it merely as a “confounder” is imprecise. A more profound perspective is that macro-factors like SES are themselves the fundamental causes (or “Common Causes”) that shape the maternal microbiome. A mother’s gut microbiome characteristics rarely exist in isolation; they are always intricately interwoven with her genetic background, dietary habits, lifestyle, socioeconomic status, psychological stress levels, and overall health. These factors are themselves powerful determinants of the fetal intrauterine environment, the quality of breast milk, and the postnatal nurturing environment. Therefore, when we observe that a certain “beneficial” microbial pattern or its metabolites are associated with better neurodevelopmental outcomes in offspring, we cannot easily conclude that the microbes are playing a causal role. A more parsimonious and more likely alternative theory is that the microbiome is merely a sensitive “biomarker” of the mother’s overall health and socioeconomic status, not a “causal mediator” driving offspring development. This cognitive upgrade is a prerequisite for an in-depth discussion of the core controversies in this field, which we will elaborate on in the next Section.

### 5.2 Challenge two: the great chasm between animal models and human reality

A large body of mechanistic insights in this field comes from germ-free or humanized animal models, especially mice. These models are undoubtedly powerful tools for exploring biological possibilities, but when extrapolating their findings directly to human neurodevelopment, one must recognize the vast chasm between the two. First, the physiological differences between species are enormous, including the maturation timing of the immune system, metabolic rates, gut structure, and most importantly, the complexity of the brain and social behavior patterns. Second, germ-free animals are themselves an extreme and highly artificial pathological state, with abnormal brain and immune system development. Whether the “restorative” effects of microbes observed in this context can represent the real situation in a normal, complex human environment is a huge question. Therefore, any conclusion derived from animal models should be regarded as a preliminary hypothesis to be validated in humans, not as established fact.

### 5.3 Challenge three: association does not equal causation—an eternal warning

The field is rife with the tendency to interpret correlational findings as causal relationships. For example, piecing together the correlation between “early milk HMO concentration” and “later cognitive scores” with the causal evidence of “butyrate promoting myelination in mice” to construct a seemingly perfect mechanistic pathway. While this practice is narratively appealing, it is not scientifically sound. In the absence of rigorous randomized controlled trials (RCTs) that can intervene in humans and observe the expected changes in neurodevelopmental outcomes, all assertions about the key roles of specific microbes, metabolites, or pathways in human neurodevelopment should be considered speculation.

## 6 Deepening the fundamental challenge: the microbiome as a biological imprint of the socioeconomic gradient

In the previous section, we upgraded the concept of “confounding factors” to “common causes.” This is not just a terminological change, but a fundamental shift in perspective. It forces us to confront a more subversive alternative theory—the “Proxy Hypothesis.” This Section aims to positively articulate and examine this hypothesis, which we believe is key to understanding the many contradictions and translational difficulties in the current field.

### 6.1 Articulating the “proxy hypothesis”: the microbiome as a biological imprint of the social gradient

The core tenet of the “Proxy Hypothesis” is that most of the “microbiome-neurodevelopment” associations observed in human studies may have very weak, or even non-existent, independent, direct causal effects. Instead, the composition and function of the microbiome are largely a direct reflection of the mother’s socioeconomic status (SES), living environment, dietary patterns, and psychological stress levels. In other words, the gut microbiome acts more like a highly sensitive biological sensor and recorder, faithfully “encoding” an individual’s life experiences into biological signals, rather than an “engineer” that can actively and independently shape long-term health outcomes.

This hypothesis is strongly supported by decades of research in social epidemiology. For example, Sir Michael Marmot’s classic work on the social gradient in health convincingly demonstrated that almost all health outcomes show a clear step-wise distribution across income, education, and occupational status (Demakakos et al., 2008). This gradient effect is not a simple case of “poverty causes disease” but a complex process driven by psychosocial factors such as autonomy, social participation, and sense of control that permeates the entire social hierarchy. These macroscopic social forces affect offspring development through two main pathways: (1) Directly shaping biology: They determine the mother’s nutritional intake, stress hormone levels (e.g., cortisol), inflammatory state, and physical and chemical exposures in her living environment, all of which profoundly affect her microbiome. (2) Shaping behavior and environment: They determine the mother’s health literacy, parenting behaviors, and the early learning environment she provides for her child.

Therefore, when a study finds that a certain “beneficial” gut microbiota feature is associated with better cognitive scores, the “Proxy Hypothesis” offers a more parsimonious explanation: mothers with this microbiota feature may themselves have higher socioeconomic status, access to better quality food, experience lower psychosocial stress, and provide a richer cognitive stimulation environment for their children. It is these factors, acting in concert, that drive better neurodevelopmental outcomes, while the microbiome is merely a “concomitant phenomenon” in this complex causal network, a measurable “biological imprint.”

### 6.2 Reinterpreting the literature: incorporating evidence from social epidemiology

Once we put on the “glasses” of the “Proxy Hypothesis,” many findings from microbiome research can be reinterpreted. For example, the controversy over cesarean section (Section 4.2) likely stems from the varying degrees to which different studies have controlled for the socioeconomic indications behind the procedure. Similarly, the failure of FMT in ASD treatment (Section 4.3) may indicate that for a nervous system already shaped by complex genetic and early environmental factors, simply changing a downstream variable like the gut microbiota is as ineffective as trying to treat pneumonia by wiping the forehead of a feverish patient.

This perspective is highly consistent with the theory of “Biological Embedding” in developmental psychology and neuroscience. The work of James P. Shonkoff and others has clearly elucidated that adverse experiences in early life (such as poverty, neglect, violence) can “carve” negative effects into an individual’s biology through epigenetic, endocrine, and neural circuit changes, thereby increasing their lifelong risk of various physical and mental illnesses (Shonkoff et al., 2009). Within this powerful theoretical framework, changes in the gut microbiome can be fully understood as one of the many pathways through which adversity is “embedded” into biology—a “result” of adversity, not a “cause.”

Ignoring this grand socio-biological context is a concentrated manifestation of the “biologism” bias in current microbiome research. It leads researchers to seek all explanations within biology, thereby inadvertently exaggerating the relative importance of the microbiome.

### 6.3 Future research directions: how to distinguish “proxy” from “cause”

Acknowledging the powerful explanatory force of the “Proxy Hypothesis” does not mean completely denying the independent causal effects of the microbiome. The real challenge is to design studies that can distinguish the contributions of these two. Future research must go beyond simple association analysis and adopt more rigorous causal inference designs:

#### 6.3.1 Mendelian randomization (MR)

Using genetic variants associated with microbiome features as instrumental variables to simulate a random allocation, thereby inferring the causal effect of the microbiome on health outcomes to some extent.

#### 6.3.2 Sibling-comparison designs

By comparing siblings raised in the same household, family-level socioeconomic and genetic confounding factors can be largely controlled, allowing for a clearer isolation of the unique effects of individual-level factors (including the microbiome).

#### 6.3.3 Natural experiments

Using social policies or environmental interventions (such as nutritional improvement programs, environmental remediation projects) as “natural experiments” to observe the extent to which these macro-interventions affect health outcomes by changing the microbiome.

Only through these more advanced research designs can we possibly answer the core question: after controlling for the powerful socioeconomic gradient, how much clinically significant, independent contribution does the maternal–infant microbiome have left?

## 7 Integration and outlook: a hierarchical socio-biological causal model

Based on the in-depth discussion of the “Proxy Hypothesis” in the preceding sections, this review ultimately discards the original “flattened” interaction network model and proposes a “Hierarchical Socio-Biological Causal Model” that better reflects real-world complexity. This model aims to clarify the asymmetry of influence among different levels of factors and to place the microbiome in its proper position within the socio-biological system. The core idea of this model is that the factors influencing offspring neurodevelopment are not on the same plane but form a hierarchical structure with clear upstream and downstream relationships.

### 7.1 Top level: systemic driving factors

At the top of the model are Socioeconomic and Environmental Factors (SES and Environment). This includes family income, parental education level, living environment, social support networks, and systemic discrimination and stress. These factors are exogenous “master regulators” that decisively shape all variables in the levels below.

### 7.2 Middle level: biological and behavioral mediators

The middle level is the concrete embodiment of how socioeconomic factors are “embedded” in biology. It contains two major interacting subsystems:

1. Maternal Physiology and Behavior: This includes the mother’s genetic background, nutritional status, metabolic health, psychological stress levels, and the overall physiological state reflected by her microbiome.
2. Postnatal Environment and Behavior: This includes breastfeeding practices, solid food introduction patterns, family parenting styles, and the cognitive stimulation and socio-emotional support provided to the infant.

In this model, the maternal microbiome is no longer an independent node parallel to SES, but is considered part of the maternal physiological state, an endogenous variable and mediating factor profoundly regulated by the top-level SES.

### 7.3 Bottom level: individual development

The bottom level consists of the proximal factors that directly influence offspring neurodevelopment, including the infant’s own genetic susceptibility and the gut microbiome established in early life.

**Figure 1:**
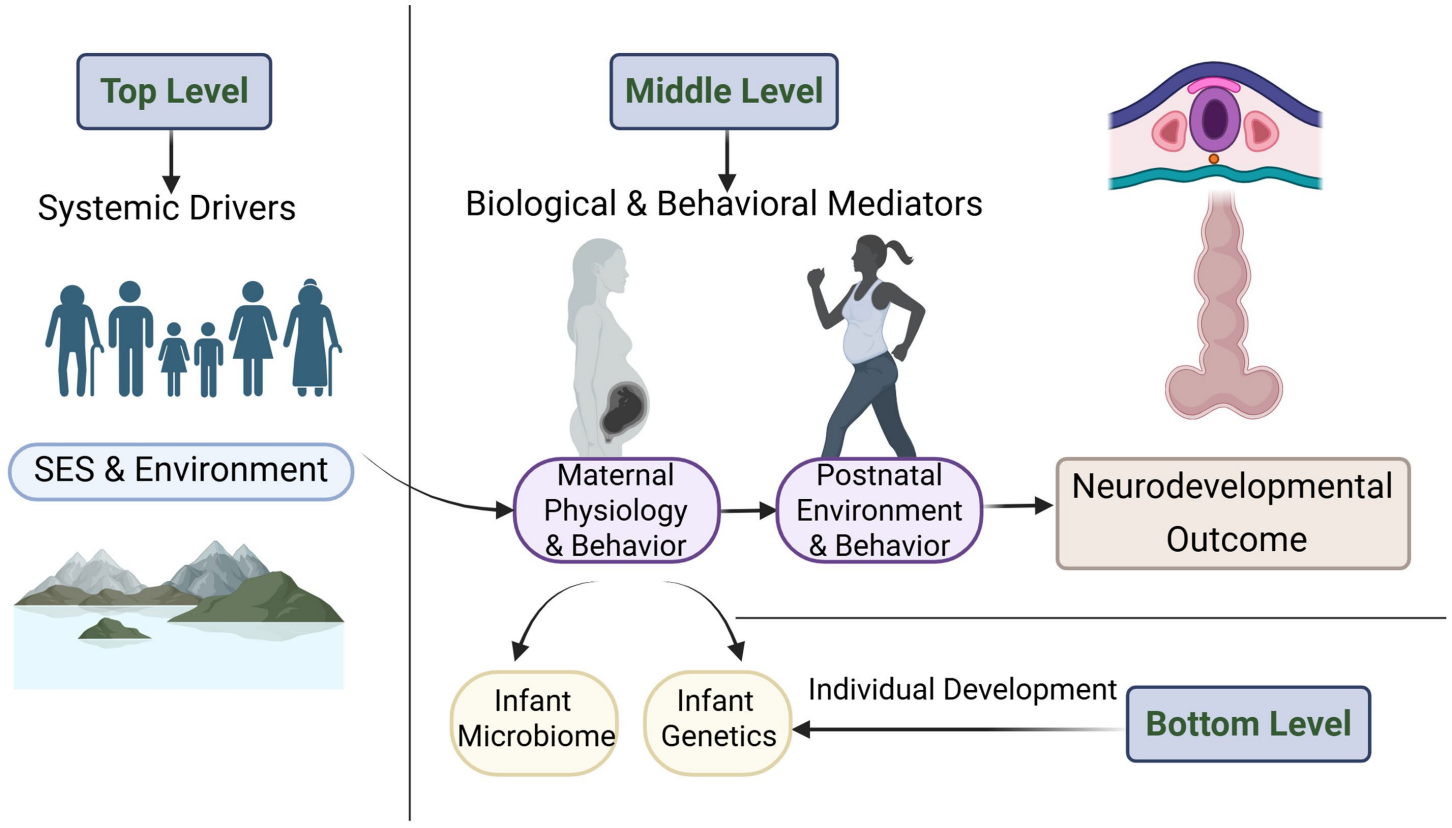
This conceptual model illustrates how socioeconomic and environmental factors (top level) shape neurodevelopmental outcomes through a cascade of biological and behavioral mediators (middle level), ultimately influencing individual-level factors such as infant microbiome and genetics (bottom level). Systemic drivers such as family income, parental education, living environment, and social stress act as master regulators that influence a range of factors, including maternal microbiome composition, nutritional status, stress levels, other aspects of maternal physiology and behavior, and postnatal care practices.

To visually represent this concept, we have constructed the following new model, as shown in Figure 1.

This hierarchical model provides us with a more cautious and realistic framework. It does not negate the potential role of the microbiome but emphasizes its constrained and mediating nature. This means that any effort to improve neurodevelopment by intervening on the microbiome, if detached from consideration of its upstream socioeconomic determinants, is likely to have minimal effect. Based on this more prudent framework, future research should shift from pursuing a single “decisive factor” to answering the following more fundamental and critical scientific questions:

1. Quantifying Mediation Effects: What proportion of the effect of socioeconomic status on neurodevelopment is actually mediated by changes in the microbiome?
2. Identifying Interactions: Does the effect of the microbiome differ across different socioeconomic contexts? Do “environment-microbiome” interactions exist, such that beneficial microbial interventions might only exert their maximum potential in more favorable social environments?
3. Mismatch from an Evolutionary Perspective: The “microbial mismatch” hypothesis resulting from modern lifestyles is highly suggestive. By comparing mother-infant microbial transmission patterns in industrialized societies with those in non-industrialized, traditional communities, can we identify key microbial functions that were crucial in human evolution but are now “lost”? Is the “loss” of these functions more severe in disadvantaged populations, thereby exacerbating health inequalities?
4. Assessing the Realism of Interventions: In real-world community settings, are interventions aimed at improving the microbiome (such as providing probiotics or dietary fiber supplements) feasible? What is their cost-effectiveness? How do their effects compare to macro-policy interventions that directly improve socioeconomic conditions?

## 8 Conclusion

Through a critical evaluation of the existing evidence in the field of maternal–infant microbiome and offspring neurodevelopment, this review arrives at a conclusion that is far more profound and cautious than the initial questions posed. We have not only critically examined the widely accepted causal pathways in the field but, more importantly, have challenged one of its foundational assumptions. The review addresses the central question raised in the introduction: to what extent does the microbiome act as an independent causal mediator, and to what extent does it serve as a biological reflection of social factors? Our final conclusion is as follows:

We propose a strong alternative theory, the “Proxy Hypothesis.” This hypothesis posits that the true driving force behind most of the currently observed “microbiome-neurodevelopment” associations is macroscopic factors such as socioeconomic status. The microbiome, in this context, acts more as a sensitive “proxy indicator” and “biological imprint” rather than an intervention target with a powerful, independent causal effect.

Based on this, we have discarded the “flattened” interaction network model and constructed a “Hierarchical Socio-Biological Causal Model” that better reflects the real-world power asymmetries. This model places socioeconomic factors at the top as “master regulators,” thereby repositioning microbiome research within a broader socio-biological framework.

However, this shift in perspective does not negate the scientific merit of the rapidly evolving microbiome field. On the contrary, the mechanistic studies conducted thus far, particularly those elucidating the roles of short-chain fatty acids (SCFAs), immune training, and the vagus nerve, have provided vital preliminary insights and a necessary biological framework for understanding how environmental signals can be transduced into neural outcomes.

Therefore, our conclusion is not that microbiome interventions (such as probiotics or HMOs) are without value, but rather that their population-level efficacy is likely constrained if the more powerful upstream social and environmental drivers are neglected.

In conclusion, this review advocates for a constructive paradigm shift: from searching for a single “magic bullet” to understanding the microbiome’s specific function within a hierarchical socio-biological system. Future research must integrate the biological rigour of mechanistic inquiries with the sociological imagination of epidemiological designs. This balanced approach is the only way to ensure that the promising discoveries in this field can eventually be translated into meaningful and socially equitable public health strategies.
